# The Present Status of Medicine and Its Relation to the Commonwealth in the Future*Read before the Niagara Co. Medical Society, by F. T. Baker, M. D., retiring President, and published by request of the Society.

**Published:** 1882-08

**Authors:** F. J. Baker


### The Present Status of Medicine and Its Relation to the Commonwealth in the Future.*

By F. J. Baker, M. D.

It will be at once seen that I have entered fields well nigh boundless. The history of medicine would make a library of itself: while preventive medicine, which is the medicine of the future, with its far-reaching laws of sanitation, would require volumes for the bare enunciation of its principles.

I shall claim then to do nothing more than touch upon some salient points as I go along, and indicate, briefly, what, it seems to me, awaits our profession in the world's to-morrow.

First, The Present Status of Medicine.

*a*, We possess but little *positive knowledge* in regard to the *nature* of disease.

Take diphtheria for illustration. Recent investigations by Klebs, Pasteur, Wood, Formad, and others, indicate that diphtheria is an infiltration of living tissue with micrococci, and that their development is intimately associated with the morbid process. Some observers have endeavored to establish an identity between diphtheria and croup, while the weight of evidence would seem to be against this affinity, since croup is proved to be a sthenic, while diphtheria is undoubtedly an asthenic disease. But while hundreds of cases have been brought forward to prove that diphtheria has been produced by a medium capable of carrying micrococci—as milk—and which, on examination, was

*Read before the Niagara Co. Medical Society, by F. T. Baker, M. D., retiring President, and published by request of the Society.

found to be filled with these parasites, Dr. Jacobi, in his recent work on this disease, comes forward and takes ground against the germ theory. And so one man, or set of men, erect a structure of experiments and place on its top the resultant fact; when along comes another investigator, with name and fame, who, with one blow topples the structure to the ground.

Perhaps instead he rears one of his own, and thereby makes "confusion worse confounded." Or very likely he leaves us without any explanation, so that we are at great loss whom to believe, or whether we had best believe at all.

But while we have no accurate knowledge concerning the nature of the causative agent of diphtheria, the weight of evidence is in favor of the theory that the disease is caused by the entrance into and the development and multiplication of micrococci or parasitic vegetable bodies in the system. Klebs and Tommasi-Crudeli claim to have discovered the minute organisms which give rise to malarial fevers. Dr. Geo. Sternberg, Surgeon U. S. A., steps forward and claims, in an independent series of investigations, conducted by himself, that the evidence upon which these investigators based their claim to a discovery, is not well founded.

Still enough has been learned to make it more than probable that the "etiology of these fevers is connected directly, or indirectly, with the presence of these organisms, or their germs, in the air and water of malarial localities."

Typhoid and typhus fevers, and scarlatina are engaging the full attention of these patient workers, and it gives great hope that the mystery of causation is to be unraveled when we make mention of such well-known names as Touissant, Koch and Lister—in addition to those already referred to—engaged in their laborious researches. But enough has been said touching our want of accurate knowledge as to the true nature of disease, particularly those we call zymotic, to make plain the sequence, viz.: *b.* That our treatment of disease at present must be largely symptomatic. Nearly every remedy in the Dispensatory has been at times vaunted as useful in diphtheria, and so of every disease known to us. This will continue so long as the specific cause is unknown. Then all will be changed; for ground will have been found upon which to base a rational and successful therapeutic course. *c.* While we are in the dark in regard to the exact *nature* of diseases, the conditions under which they are developed, are quite largely known, and are, therefore, preventable. Under the application of efficient sanitary laws we learn that in Memphis the decrease in deaths from infectious diseases during '81 as compared with '80, was 73 per cent.; in Chicago, 1.12; in Cincinnati only 48 per cent.; while in New York, Brooklyn and Philadelphia, where sanitation has not received much attention of late, the *increase* of deaths stood respectively 2.06 per cent., 1.65 per cent., and 2.68 per cent.; in Memphis during the first quarter of '80 the death-rate from diarrhœal diseases was 1.19 per 1000; for the same period of '81 it was 0.59, a decrease of 50 per cent. What a contrast with the death-rate of that city (Memphis) for the three years previous to 1878 when the yellow fever epidemic prevailed there! —and all this brought about by restoring that city on sound sanitary principles. It is hardly probable that the scourge of 1878 will be repeated so long as these principles are enforced. We are rarely called upon to see a case belonging to the infectious class, but that a careful investigation will reveal the source of the infection, and the practical point of this is that in nearly every instance a proper regard to sanitary laws would have prevented such disease. To sum up then in brief this part of my subject, we would say that, as physicians, we do not as yet possess an intimate acquaintance with the active causative agents of infectious diseases. It is likely that diphtheria and scarlatina are caused by minute organisms, called micrococci, and that they are produced as the direct result of vegetable decomposition.

It is highly probable that typhus and typhoid have their origin also in an infectious animalculæ—the bacilli. One party holds that these originate in the decomposition of organic substances, and the other that they originate in the decomposition of these substances only when these decomposing substances are mixed with the germs of the specific typhus or typhoid poison, and when these germs grow and multiply, While it is held that the specific poison giving rise to typhus and typhoid, is quite identical in nature, why so great difference in its mode of manifestation?

Whoever touches or comes near to a case of typhus is in danger of contagion. The greater number of physicians and attendants who care for such invalids are themselves attacked by the disease—while, as a matter of fact, typhoid, in reality, is never directly transmitted from person to person. Physicians and nurses who take care of typhoid patients are no more frequently attacked with the disease (according to Liebermeister) than are persons who have never seen such cases. No one has yet been able to demonstrate any distinctive difference between the parasite giving rise to diphtheria on the one hand, and scarlatina on the other, but they bear no relation to each other only that both are alike highly infectious. If Klebs and Tommasi-Crudeli have failed satisfactorily to prove that some of the organisms found in the swamp-mud and gutter-water about New Orleans are the true bacillus malariæ—and which Klebs succeeded in cultivating—and if they *have* failed to show conclusively that these organisms, when injected beneath the skin of a rabbit, give rise to a malarial fever corresponding with the ordinary paludal fevers to which man is subject; I say if they have not succeeded in demonstrating all this, they have, without doubt, opened the door to a series of investigations which will result in a correct solution of the problem. We know not yet the nature of the contagious principle of measles. Shall we accept the view of Home, Speranza, Bufalini, Locatelli, Rossi, Mayr, Wendt and a host of others, who hold that in this disease cocci exist in the blood, tears, saliva, mucus from the nose, and even in the branny desquamation of the skin; and that by inoculation with either of these, the disease may be transmitted like small-pox?

Or shall we hold with Salisbury, who ascribed measles to a fungus spore found upon decaying straw; and who succeeded in producing an exanthem bearing a close resemblance to measles by inoculations with this parasite? May not both, or all, be quite right?

The answer will come at some future time.

Let us now consider for a few minutes the second division of the subject: "The Relation of Medicine to the Commonwealth of the Future."

Without doubt the future of medicine is glorious. Its past has been most honorable, as step by step it has emerged from the mists of ignorance and superstition. But its readings and its working have been in the dark; still light has been let in upon its path all along its history, and in its gleams wonderful strides have been made toward the dawn. Its present is indeed grand as in laboratory and clinic patient workers, in every land and clime, are tugging at this mighty problem of disease—its cause, and how its deadly onslaughts may be met.

But it remains to the future to realize the mission of medicine in all its lofty conceptions.

Some investigator is to tell us, by and by, whether zymotic diseases have their origin in special animal or vegetable growths. He is going to produce these growths artificially, outside the body. And then he will take them to his laboratory, and by repeated experiment finally decide upon some drug, capable of destroying their vitality. It will then remain for extended clinical research to determine whether or not this parasite will still retain its destructive power when it meets these organisms in the system, surrounded and influenced, as they must be, by all the conditions they find therein. Salicylic and carbolic acid, and other disinfecting agents, will destroy these animalculæ, but the system cannot bear them in doses sufficiently large to act effectually as physiological antidotes. The reproduction of these low organisms is so rapid that if the acids above mentioned should "be administered internally, as often, and in doses sufficient to fulfill their indication, the remedy would become almost more destructive and dangerous to the system than the infectious animalculæ, the micrococci, or the bacilli." But here we have to thank Klebs for an important discovery. "He found that the benzoate of sodium or of magnesium destroys the bacilli of typhoid fever, while, in very large doses, it does not act deliteriously on the system." He proved the efficacy of the remedy, or specific, in the case of one of the workmen in his own laboratory, who was taken sick with typhoid from exposure to the bacilli, while being occupied with their culturing. Nine days treatment with the benzoate of sodium in the form of injection, mouth-wash, gargle, by inhalation and by internal administration, in doses of about 20 grs. every two hours, caused the fever, with every other symptom totally to leave him.

About 320 grams of the remedy were given daily to the patient by the different modes of its application. The only sequela of which the patient complained, was a rather remarkable weakness for several weeks.

Without doubt the near future will bring to us antidotes—perfectly harmless to the different structures of the body—fully competent to destroy the various parasites giving rise to zymotic diseases. The following colloquy from *Punch* has much of truth, as the future will reveal it, running through its humor, (it represents a customer at the counter of the chemist.) Says customer: "My nephew is just starting for Sierra Leone, and I thought I could not make him a more useful present than a dose of your best yellow fever. Would you tell me the price, please?" Chemist: "Well, m'am, the germs are so difficult to cultivate in Europe that I would advise your waiting for the next West Indian mail, when I am expecting a nice fresh consignment from St. Thomas. Meanwhile we could recommend our half-guinea traveler's assortment of the six commonest zymotics, and could add most of the tropical diseases from stock at five shillings each. We have some nice Asiatic cholera, just ripe, but they are more expensive."

Specific medication will not then be a chimera, nor will he who professes and practices it be dubbed a charlatan. But a higher position than this awaits the physician of the future. As the war-horse scents the battle afar off, so will the keen-eyed, alert physician discern in earth and air, and sky, portents of disease. His words of warning and advice will reach every hearth-stone where he reigns as arbiter of health, so that back of effect, and back of cause the antidote may be applied. Censure will surely attach to him if disease invades before the warning comes.

As the physician justly merits, and quite generally receives discharge, who fails to recognize a well-marked, familiar disease, *then* his services will be no longer wanted if he fails to *anticipate* disease. I do not *know*, but I think that in that truer time to be a doctor so-and-so, will be honor e.iough for any man. No *ic* or *pathy* will be appended to add luster to his name or calling. Potencies, nor active principles, nor mineral, nor herb shall draw invidious lines. In solid phalanx shall the healers stand. Death will not then claim one-third the race before the age of five. Schools and colleges will not then be approved and ingeniously applied machines where health and vigor is crowded out as knowledge is crammed in.

*Ex*tensive life will grow at the expense of the *in*tensive. Physiology and hygiene will be taught and understood. *Then* a sound preliminary education will be required of each applicant at the threshold of our medical colleges. The term of study will be extended and the course thoroughly graded. The different chairs will be well endowed, and it is more than likely that all temptation to the granting of degrees will be removed from the schools and vested in State Boards.

This change is not to be brought about in a day, nor by a feeble, spasmodic allegiance to a code of ethics; nor by revolutionary measures relating to such code; but in years patiently spent in attaining to a high mental, moral and professional excellence.

